# Retraction: Efficacy of Concomitant Therapy Versus Triple Therapy in Eradicating Helicobacter pylori Infection: A Retrospective Study

**DOI:** 10.7759/cureus.r229

**Published:** 2026-05-14

**Authors:** Muhammad Umair Mazhar, Hafiz Muhammad Ahmad Anees, Samana Mukhtar, Humna Irshad, Ayman Irshad, Abdul Moizz, Abdul Eizad Asif, Hassan Durrani, Haiqa Asif, Hannan Hashmat, Muhammad Khizer Azeem

**Affiliations:** 1 Stroke Medicine, University Hospital Coventry and Warwickshire NHS Trust, Coventry, GBR; 2 Internal Medicine, Allama Iqbal Medical College, Lahore, PAK; 3 Internal Medicine, Hayat Memorial Teaching Hospital, Lahore, PAK; 4 Internal Medicine, Kishwar Fazal Teaching Hospital, Lahore, PAK; 5 Internal Medicine, Shalamar Medical and Dental College, Lahore, PAK; 6 Internal Medicine, Rahbar Medical and Dental College, Lahore, PAK; 7 Internal Medicine, Rashid Latif Medical and Dental College, Lahore, PAK; 8 Internal Medicine, Rawalpindi Medical University, Rawalpindi, PAK

This article has been retracted by the Editor-in-Chief due to concerns regarding the reliability of the reported data, analyses, and aspects of the study methodology. Post-publication review identified issues affecting confidence in the integrity and verifiability of reported findings. The authors agree with the decision to retract.

